# Co-occurrence network analysis reveals the alterations of the skin microbiome and metabolome in adults with mild to moderate atopic dermatitis

**DOI:** 10.1128/msystems.01119-23

**Published:** 2024-02-06

**Authors:** Paulo Wender P. Gomes, Helena Mannochio-Russo, Junhong Mao, Haoqi Nina Zhao, Jacob Ancira, Craig D. Tipton, Pieter C. Dorrestein, Min Li

**Affiliations:** 1 Collaborative Mass Spectrometry Innovation Center, Skaggs School of Pharmacy and Pharmaceutical Sciences, University of California San Diego, La Jolla, California, USA; 2 Colgate−Palmolive Company, Piscataway, New Jersey, USA; 3 RTL Genomics, MicroGenDX, Lubbock, Texas, USA; 4 Department of Pediatrics, University of California, San Diego, California, USA; Katholieke Universiteit Leuven, Leuven, Belgium

**Keywords:** microbiota, skin inflammation, metabolomics profiling, dysbiosis, pathogenesis

## Abstract

Skin microbiome can be altered in patients with atopic dermatitis (AD). An understanding of the changes from healthy to atopic skin can help develop new targets for treatment by identifying microbial and molecular biomarkers. This study investigates the skin microbiome and metabolome of healthy adult subjects and lesion (ADL) and non-lesion (ADNL) of AD patients by 16S rRNA gene sequencing and mass spectrometry, respectively. Samples from AD patients showed alterations in the diversity and composition of the skin microbiome, with ADL skin having the greatest divergence. *Staphylococcus* species, especially *S. aureus*, were significantly increased in AD patients. Metabolomic profiles were also different between the groups. Dipeptide derivatives are more abundant in ADL, which may be related to skin inflammation. Co-occurrence network analysis of the microbiome and metabolomics data revealed higher co-occurrence of metabolites and bacteria in healthy ADNL compared to ADL. *S. aureus* co-occurred with dipeptide derivatives in ADL, while phytosphingosine-derived compounds showed co-occurrences with commensal bacteria, for example*, Paracoccus* sp., *Pseudomonas* sp., *Prevotella bivia*, *Lactobacillus iners*, *Anaerococcus* sp., *Micrococcus* sp., *Corynebacterium ureicelerivorans*, *Corynebacterium massiliense*, *Streptococcus thermophilus*, and *Roseomonas mucosa*, in healthy and ADNL groups. Therefore, these findings provide valuable insights into how AD affects the human skin metabolome and microbiome.

**IMPORTANCE:**

This study provides valuable insight into changes in the skin microbiome and associated metabolomic profiles in an adult population with mild to moderate atopic dermatitis. It also identifies new therapeutic targets that may be useful for developing personalized treatments for individuals with atopic dermatitis based on their unique skin microbiome and metabolic profiles.

## INTRODUCTION

As the largest organ of the human body, the skin plays a vital role in maintaining a stable internal environment and protecting the body from external factors. The skin’s outer layer is composed of lipids and proteins, as well as skin appendages like hair follicles and eccrine glands, which produce lipids, antimicrobial peptides, enzymes, and salts (1). When the skin is stressed, such as in dermatitis, changes may occur in the microbial community living on the skin and the molecules produced by skin cells.

It is well established that the skin hosts microbes that are essential in the systemic, pathophysiologic, and biochemical balance of the organism (2). These interactions are critical for health and commonly noticed in the human skin (3). Dermatitis and other stress on the skin can change the molecules derived from the host microbes and disrupt the healthy balance between the host and skin microbiome. Also, inflammatory conditions significantly impact the skin barrier function, resulting in increased permeability and water loss (4). This disruption has the potential to modify the skin’s chemical composition by perturbing the levels of essential lipids, including ceramides, cholesterol, and free fatty acids, which play a critical role in maintaining the integrity of the barrier (4). Furthermore, inflammatory processes give rise to reactive oxygen species that can exert an influence on the chemical composition by inducing damage to cellular structures, lipids, and proteins (5). It should be noted that since inflammatory conditions disturb the microbiome, it can also lead to dysbiosis. Dysbiosis refers to an alteration in the composition of microbial communities, which can have significant implications for skin health and its function (6). For instance, conditions, such as atopic dermatitis, have been closely associated with a reduction in microbial diversity and an overgrowth of specific pathogenic bacteria, such as *S. aureus* (7, 8).

**Fig 1:**
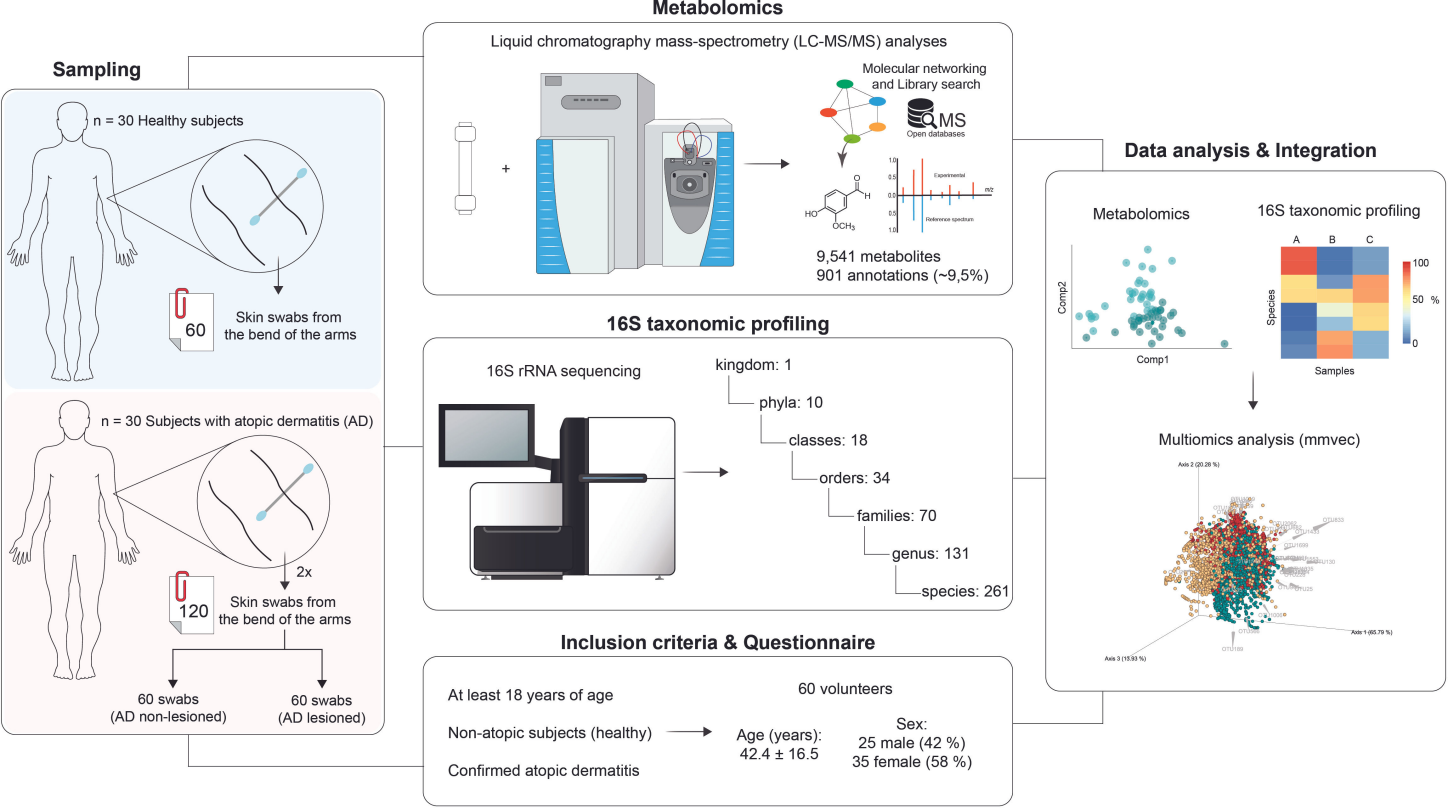
Experimental design and analytical workflow. A total of 60 volunteers, selected based on the inclusion criteria, participated in this study. Consequently, we collected 180 skin swabs as follows: 60 swabs from 30 healthy subjects and 120 swabs from 30 subjects with atopic dermatitis (60 from nonlesioned areas and 60 from areas with lesions). Out of the total, 90 swabs were used for 16S rRNA gene-sequencing analysis, and 90 were used for metabolomics analysis using liquid chromatography-mass spectrometry. The metabolomics data were analyzed by molecular networking approach and library search. A multi-omic microbe-molecule co-occurrence analysis (microbial-molecule vectors [mmvec]) was used to combine both data sets.

In recent years, advancements in mass spectrometry and 16S ribosomal ribonucleic acid (rRNA) gene amplicon-sequencing technologies have expanded our understanding of the microbes residing in the human skin (9–12), which also influence the molecules present on the skin (13). Consequently, mass spectrometry-based metabolomics and 16S rRNA sequencing have garnered increased attention in skin research (9–16). Metabolomics focuses on the analysis of small molecules produced by cells, tissues, or organisms and how they respond to various conditions or stress (17–19). In contrast, 16S rRNA sequencing examines one or two small regions of the gene material from microorganisms (20). This approach enables providing a comprehensive view of microbial communities in biological samples. Thus, the combination of these two approaches has proven valuable in identifying changes in the metabolic and microbiome profiles of various biological samples (21, 22). Therefore, in this study, we used metabolomics and 16S rRNA sequencing approaches to investigate the changes in the metabolome and microbiome of skin swabs of healthy patients and subjects with atopic dermatitis (Fig. 1). Furthermore, the two omics data were integrated by co-occurrence network analysis to explore the relationship of metabolites and bacteria in relation to atopic dermatitis.

## RESULTS

### Microbiome profiles by 16S rRNA sequencing

**Fig 2:**
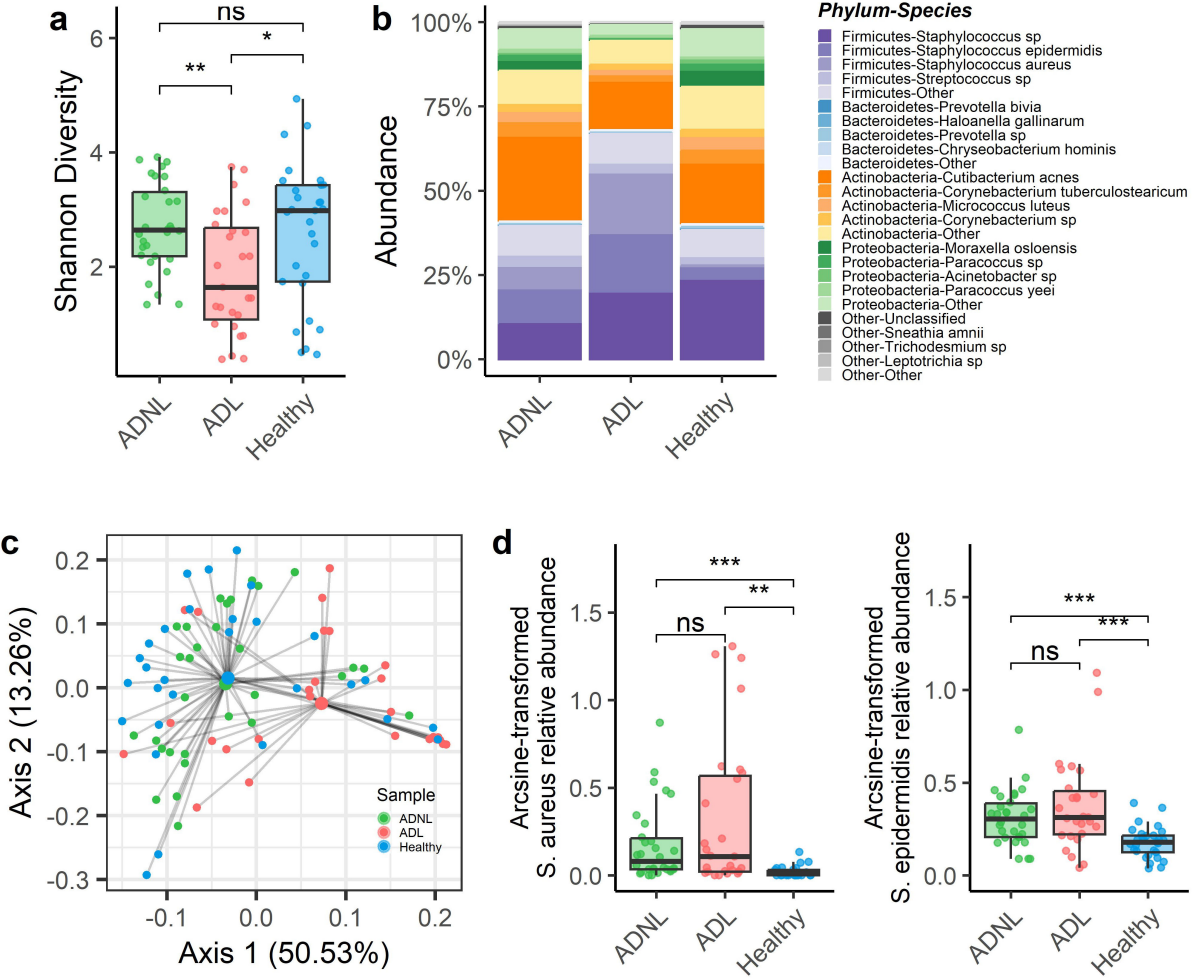
The skin microbiome profiles of lesion and nonlesion of atopic dermatitis patients and healthy subjects characterized by 16S rRNA gene sequencing. (**a**) Shannon diversity of 16S rRNA sequencing, grouped and colored by the three sample groups: health, ADNL, and ADL. Boxplots show the median (designated by the horizontal line), first, and third quartiles of each of the three groups. Additionally, analysis of variance (ANOVA) and Tukey testing was conducted and showed samples to significantly differ between groups, as well as ADL arguing the most between the two other sample groups. (**b**) Relative abundance of the top species grouped and colored according to phyla. (**c**) PCoA plot based on weighted Unifrac distances of 16S microbiome data. Axes represent the summarization of variability in the data set; the greater the distance between points, the greater the dissimilarity. Sample grouping was found to explain significant variation in overall bacterial dissimilarity (PERMANOVA; *P* = 0.001, *R*^2^ = 0.13). Larger points indicate each group’s centroids, with black lines connecting each point with the corresponding centroid. (**d**) The arcsine-transformed relative abundance of *S. aureus* and *S. epidermidis* was grouped and colored by the sample groups. Both species were indicated to be differentially abundant per ANCOM-BC with pairwise testing by Tukey’s honestly significant difference test. Significance asterisks represent pairwise testing, where ns = *P* > 0.05; *0.01 < *P* < 0.05; **0.001 < *P* < 0.01; and ***P < .001.

Atopic dermatitis significantly changed the skin microbial composition. The alpha diversity (Shannon diversity index) of the AD lesion was significantly lower than the AD nonlesion (*P* < 0.01) and healthy group (*P* = 0.01; Fig. 2a), while the diversity of AD nonlesion and healthy groups was not significantly different. The overall microbial profiles of each group at the species level are illustrated in Fig. 2b. The most abundant bacterial lineages observed included *Cutibacterium acnes*, *Staphylococcus* sp.*/epidermidis/aureus*, and *Corynebacterium tuberculostearicum*, accounting for 59% of all read counts study-wide. Beta diversity was assessed using weighted UniFrac distance to summarize the microbial composition between the groups. Principal coordinates analysis (PCoA) was used for qualitative clustering and permutational multivariate analysis of variance (PERMANOVA) for significance testing (Fig. 2c). Though there was some overlap in the bacterial profiles observed, the mean bacterial composition of AD lesion samples was significantly shifted from AD non-lesion (pairwiseADONIS; adj.*P* = 0.002, *R*^2^ = 0.19) and healthy samples (adj.*P* = 0.002, *R*^2^ = 0.11). Then, analysis of compositions of microbiomes with bias correction (ANCOM-BC) was used to identify six species-level clusters that significantly differed between groups (Table S2). Of these, two species (*S. aureus* and *S. epidermidis*) were found at significantly greater proportions in AD and adult subjects and lesion (ADNL) samples compared to healthy skin (Fig. 2d).

### Metabolomics molecular profile

**Fig 3:**
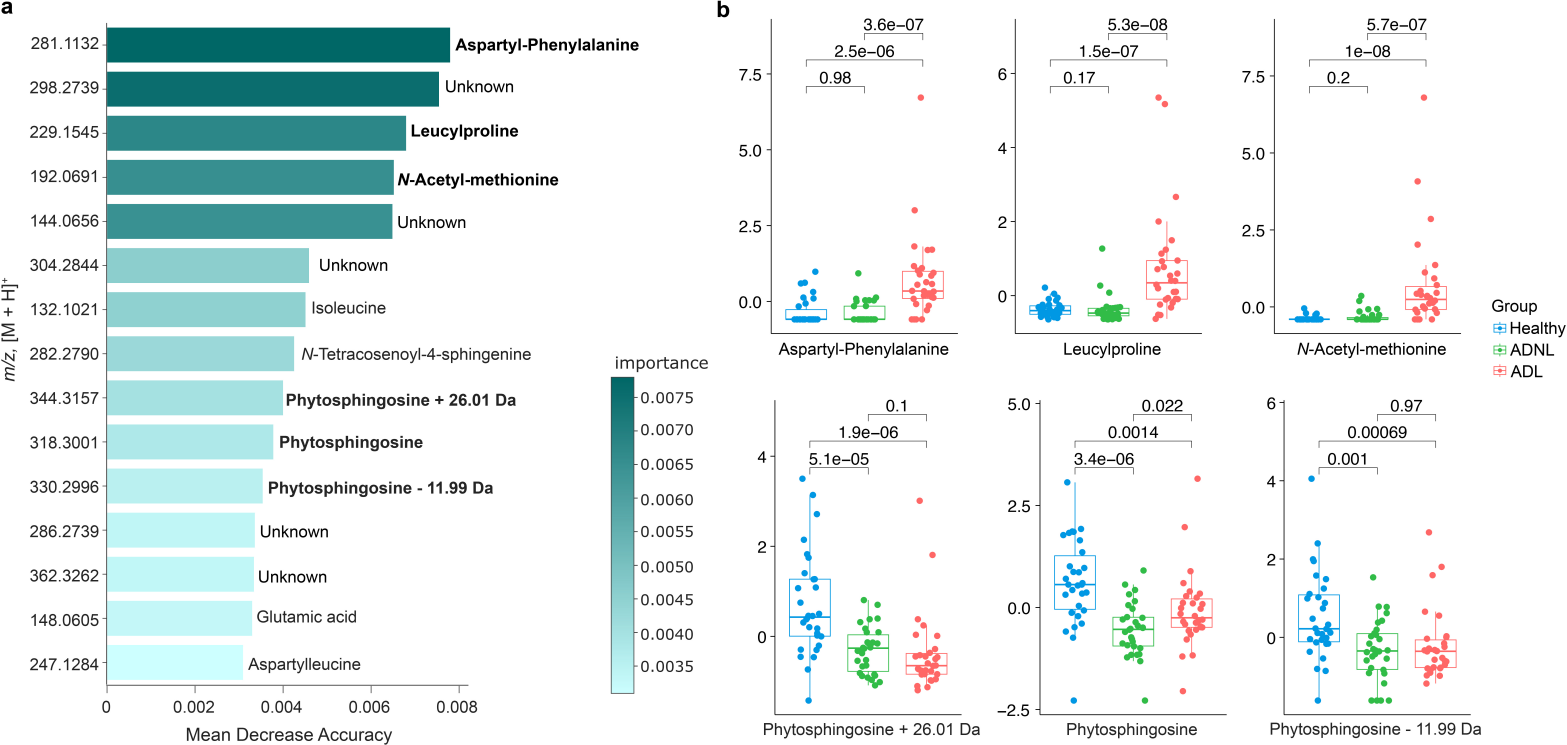
Mapping the main molecules in healthy and atopic dermatitis skin samples. (**a**) The barplot contains the top 15 MS features that are differentiated from three groups: healthy, ADNL, and ADL. They were classified by random forest analysis. The MS features were ranked by mean decrease accuracy. The features with annotations that stood out with higher concentration in ADL and healthy groups (in bold) were selected to build boxplots. (**b**) The *y*-axis represents the normalized peak intensity per MS feature, and the *x*-axis represents the boxplots and their distribution of values for a specific MS feature by sample group. The *P* values < 0.05 indicate significant differences between groups. Aspartyl-phenylalanine (*m/z* 281.1132), leucylproline (*m/z* 229.1545), and *N*-acetyl-methionine (*m/z* 192.0691) were detected in the unhealthy (ADL) group. In contrast, phytosphingosine (*m/z* 318.3001) and two related compounds (*m/z* 344.3157 and 330.2996) were detected in higher concentrations in the healthy group.

Overall, the processed mass spectrometry data resulted in 9,541 MS^1^ features (i.e., a detected signal with *m/z* and retention time corresponding to a detected molecule). MS^2^ spectra were collected for all MS^1^ features, which were represented in a molecular network (Fig. S1a) based on spectral similarity (23). Around 9.5% of the MS^2^ spectra were annotated by library matches against the reference Global Natural Product Social Molecular Networking (GNPS) public libraries, which is similar to the ~10% annotation rate reported for other human matrices (24). Some examples of annotated molecules are illustrated in Fig. S1b. It is important to note that the suspect library (25), an *in silico* tandem mass spectrometry (MS/MS) library utilized to propagate annotation on GNPS, accounted for around 80% of the annotations. We used the MS features to create pairwise partial least squares (PLS)-discriminant analysis models. Fig. S2a and b) show a remarkable separation between healthy vs ADNL, as well as between healthy vs adult subjects and lesion (ADL), suggesting that the metabolic profiles of both ADNL and ADL are distinct from the healthy group. For this, we employed a method called random forest classifier and trained it on the data set using cross-validation. By measuring the decrease in classification accuracy when each feature was randomly permuted, we filtered the 15 most informative features for distinguishing between the groups (Fig. 3a). Then, we annotated three out of the top five molecules that contribute to classifying different groups, such as aspartyl-phenylalanine, leucylproline, *N*-acetyl-methionine, and others more abundant in ADL samples. Five of the top 15 metabolites were not annotated, and it can be attributed to limited MS/MS databases or even to novel molecules. To visualize the results, we created boxplots (Fig. 3b) to visualize the distribution of the normalized peak intensities of six features across the different groups (healthy, ADNL, and ADL). The significant differences in relative concentration for these molecules per group were assessed by ANOVA analysis (*P*-value < 0.05). Aspartyl-phenylalanine, leucylproline, *N*-acetyl-methionine, phytosphingosine, and two related compounds were annotated at level 2 by GNPS libraries.

### Microbiome and metabolomic integration

**Fig 4:**
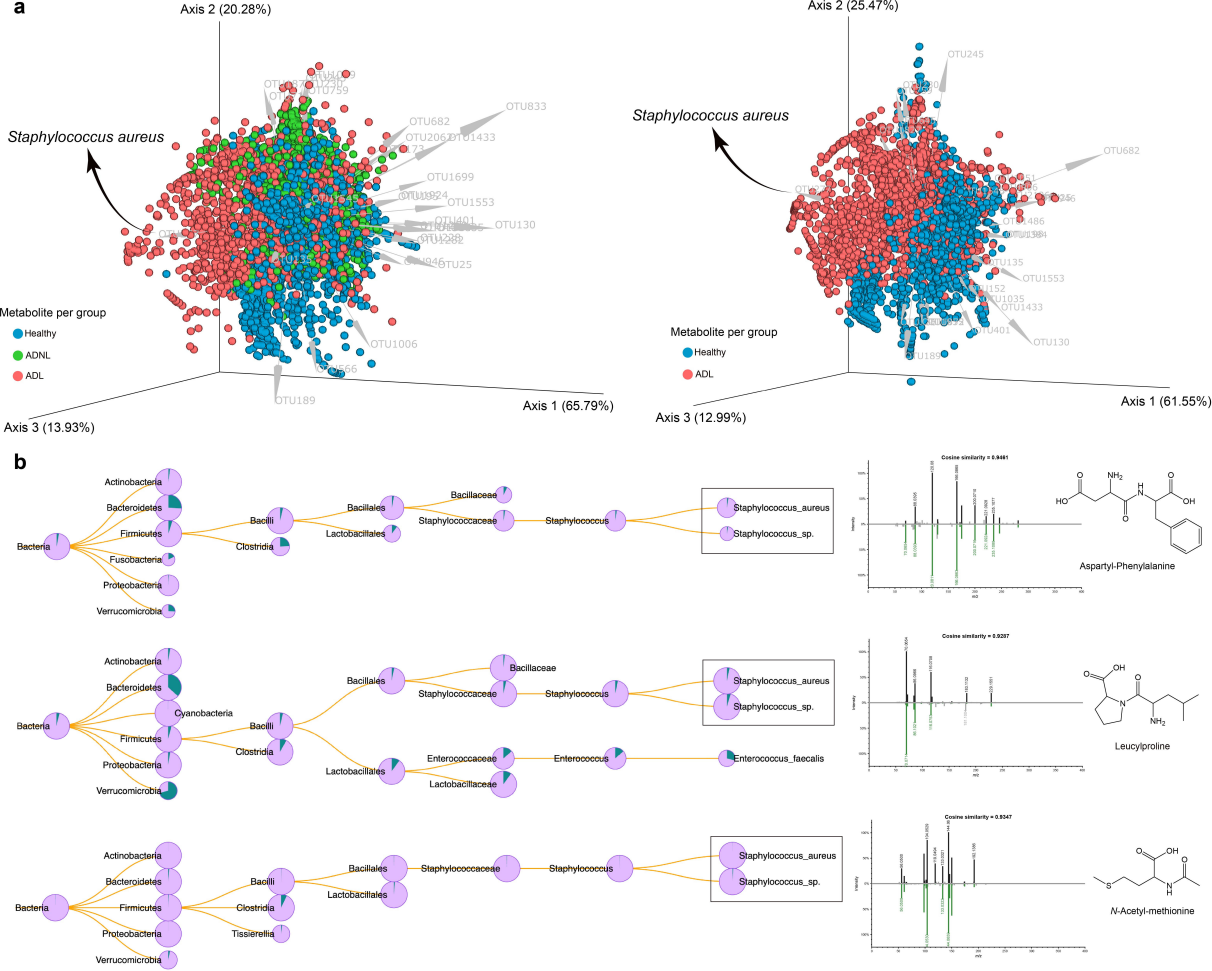
Microbe-molecule co-occurrence biplot. (**a**) mmvec analysis included samples from the healthy, atopic dermatitis without lesion, and atopic dermatitis with lesion groups (90 samples). Spheres in the biplot represent the molecules, while the arrows represent the microbes. Spheres were colored based on which group each molecule was most abundant in, while microbes were colored based on which species they belonged to. Small angles between arrows indicate microbes co-occurring with each other. Similarly, closer spheres indicate molecules co-occurring. Arrows pointing toward a group of molecules indicate microbe-molecule co-occurrence. This biplot shows the 30 most important OTUs (higher vector magnitude). (**b**) microbeMASST search outputs confirm that monocultures of *S. aureus* and *S*. sp. could produce molecules, such as aspartyl-phenylalanine, leucylproline, and *N*-acetyl-methionine, which were detected in higher abundance in ADL (retrieved from random forest analysis). Pie charts display the proportion of MS/MS matches found in the deposited reference database. Green indicates a match with the monocultures, while pink represents a nonmatch.

The integration of 16S rRNA sequencing results with metabolomics data using the mmvec (26) method enabled the prediction of co-occurrence patterns between molecules, microbial species, and sample groups. High co-occurrence probabilities indicate potential relationships, such as a microbe producing a specific molecule or a molecule inducing the proliferation of a specific microbe. The biplot in Fig. 4 visually represents these microbe-molecule co-occurrences. We observed higher co-occurrence probabilities in healthy and ADNL groups compared to ADL, represented by more bacterial operational taxonomic units (OTUs) toward healthy and ADNL groups (Fig. 4a). In the ADL group, a correlation was observed with the presence of *S. aureus*, which co-occurred with specific molecules, such as aspartyl-phenylalanine, leucylproline, and *N*-acetyl-methionine, previously described in higher concentration in ADL. This suggests a potential association between *S. aureus* and the production or induction of these molecules in ADL. Furthermore, microbeMASST (27) was employed to make MS/MS single searches for the molecules detected in higher abundance in the ADL group (Fig. 4b). microbeMASST is a database where users can search MS/MS spectra previously detected in experimental data from bacterial, fungal, or archaeal monoculture extracts (27). In that regard, aspartyl-phenylalanine, leucylproline, and *N*-acetyl-methionine were also detected in strains of *S. aureus*, and it is a piece of evidence that these molecules could be produced by *S. aureus* strains. Also, these findings are according to the findings observed in the diversity of the skin microbiome (Fig. 2d), which showed a high abundance of *S. aureus*.

On the other hand, bacteria mainly present in healthy and ADNL groups, such as *Paracoccus* sp.*, Pseudomonas* sp.*, Prevotella bivia, Lactobacillus iners, Anaerococcus* sp.*, Micrococcus* sp.*, Corynebacterium ureicelerivorans, Corynebacterium massiliense, Streptococcus thermophilus,* and *Roseomonas mucosa* (see Fig. S3) showed associations with phytosphingosine of *m/z* 318.3001 and two related compounds of *m/z* 344.3157 and *m/z* 330.2996, respectively (Fig. S1b). The 30 most important OTUs , their taxonomy, and the molecules’ information were retrieved from the mmvec analysis (*m/z*, retention time, and spectral library annotations if available). For more details, mmvec per pair analyses can be found in Fig. S3. An alternative visualization of these co-occurrences is shown in Fig. S4 to S6, in which these top 30 OTUs with co-occurrence probabilities above 5.0 are depicted (Tables S3 to S5). Therefore, these results provide valuable insights into the co-occurrence patterns between microbiota and metabolites in different groups, shedding light on potential microbial–metabolite interactions and their relevance in the context of skin health.

## DISCUSSION

In this study, to understand the underlying impacts of atopic dermatitis on human skin, we investigated the microbiome and metabolomic profiles of skin swab samples from healthy adult subjects and patients with mild to moderate atopic dermatitis using rRNA sequencing mass spectrometry-based metabolomics. Our results demonstrated significant alterations in the skin microbiome, in some comparisons between healthy and AD patients, or between ADL samples vs nonlesion and healthy skin. For example, diversity and overall composition were significantly altered in AD lesions but were not apparently different between healthy and lesion-adjacent (ADNL) skin (Fig. 2a through c). The reduced diversity may indicate a loss of beneficial microbial species and a shift toward a community state dominated by potentially pathogenic microbiota (28, 29). The reduced diversity and shifted composition together support that dysbiosis occurs in atopic dermatitis lesions (11, 28); however, the changes between healthy skin and lesion-adjacent skin measurements are relatively modest here.

Considering changes that were significant overall between healthy and dermatitis individuals, the greatest difference observed was due to increases in *S. aureus* and *S. epidermidis* in AD patients (Fig. 2d), regardless of whether the sample was taken from a lesion. The current findings in an adult population are consistent with previous studies, primarily in pediatric patients, that have reported the overgrowth of *Staphylococcus* species in atopic dermatitis lesions, suggesting their potential role in disease pathogenesis and exacerbation, particularly dominated by *S. aureus* strains (11, 28, 30). *S. epidermidis* is a well-known beneficial bacterium on the skin surface. It produces antimicrobial peptides to inhibit pathogenic bacteria, e.g*., S. aureus* (31), produces ceramides to maintain the skin barrier function (32), and regulates host immunity (33). However, we detected that *S. epidermidis* is also significantly increased in AD lesions. The increase in *S. epidermidis* could be due to a compensatory or antagonistic mechanism to control *S. aureus* (7, 31–33). The microbial profiling analyses indicate that active lesions in atopic dermatitis are associated with a distinct microbiome composition, and key species found in lesions are also more abundant in the adjacent skin, highlighting the importance of the skin microbiota in the pathogenesis of the disease.

Additionally, the skin metabolomic profiles were also different between the groups. The top 15 features most important for the classification were detected in high relative abundance in ADL samples. Among these molecules, three matches against GNPS spectral libraries were retrieved, i.e., aspartyl-phenylalanine, leucylproline, and *N*-acetyl-methionine. Aspartyl-phenylalanine is a dipeptide metabolic byproduct of aspartame (*N*-L-α-aspartyl-L-phenylalanine 1-methyl ester). However, there is no clear connection between consumption of aspartame and atopic dermatitis. In the same way, insights into leucylproline are also not established yet. A suitable hypothesis is that they could be coming from the degradation of proteins in the human skin and could be acting as the first line of defense against infections (34, 35). To gain more information about where these molecules are coming from, we searched its MS^2^ spectra in microbeMASST, which revealed that they could be produced by cell culture of *S. aureus* and *Staphylococcus* sp. These findings align with the higher abundance of *S. aureus* observed in atopic dermatitis patients and suggest that aspartyl-phenylalanine, leucylproline, and *N*-acetyl-methionine may originate from the skin microbiome. Thus, it indicates the influence of the skin microbiome on the metabolome when submitted to the status of atopic dermatitis.

Co-occurrence analysis between metabolomics and microbial sequencing data provided insights into possible interactions between the microbiome and human skin molecules. The decrease in crosstalk between the microbiome and metabolites in the ADL group observed in our study provides further evidence of dysbiosis in skin microbiome in subjects with ADL, highlighting the importance of microbial-metabolite interactions in skin health. Most importantly, we identified specific molecules exclusively present in the normal skin or AD lesion samples suggesting their potential roles in skin health. In AD lesions, more microbial-derived metabolites, e.g*.,* dipeptide derivatives, were detected, which may be related to skin inflammation, indicating the contribution of skin microbiome, especially *S. aureus,* to the pathogenesis of atopic diseases. While in the normal skin more host-derived metabolites were detected to correlate with the skin microbiome, such as phytosphingosine (PHS), and two related compounds, phytosphingosine is a natural lipid present in the intercellular spaces of the stratum corneum of the skin. It is one of the fundamental components of maintaining the skin barrier function (36). PHS has also been described as with anti-microbial and inflammatory activity (37, 38). The association between PHS and normal skin microbiome indicates that the crosstalk of the bacteria and molecules contributes to the maintenance of skin homeostasis and protects against atopic dermatitis progression. These identified molecules and/or bacteria could be used as new therapeutic targets for personalized treatment for atopic dermatitis. However, the correlation between the microbiome and metabolomics identified in this study is based on computational prediction (mmvec). More research is needed to elucidate the exact functions and mechanisms of these molecules in the context of skin health and AD.

Overall, our study provides valuable insight into changes in the skin microbiome and associated metabolomic profiles. It also identifies new therapeutic targets that may be useful for developing personalized treatments for individuals with atopic dermatitis based on their unique skin microbiome and metabolic profiles.

## MATERIALS AND METHODS

### Chemicals

Liquid chromatography-mass spectrometry (LC-MS)-grade acetonitrile (ACN) and water with 0.1% formic acid used for LC-MS analyses were acquired from Thermo Fisher Scientific (San Diego, CA, USA). For the extraction process, ethanol and water were supplied by Thermo Fisher Scientific (San Diego, CA, USA), all of high-performance liquid chromatography grade. Analytical-grade chemicals were purchased from Sigma-Aldrich (Steinheim, Germany).

### Study design and sample collection

The clinical study was conducted by ProDERM (Schenefeld, Germany). An independent ethics committee approved the study. All the subjects were above the age of 18 years and signed informed consent forms. The patients with mild-to-moderate AD (*N* = 30) in the bend of the arm and non-atopic healthy subjects (*N* = 30) were recruited. Inclusion criteria for AD patients included self-reported AD diagnosis with either two active lesions of a size of at least 5 cm in diameter and a local scoring atopic dermatitis (SCORAD) index (39–41) of at least 4 in each bend of the arms, or one lesion of a size sufficient to take two swabs (each 5 cm in diameter at least) and a local SCORAD index of at least 4 in the bend of the arms on sample collection day. Exclusion criteria for all subjects included systemic therapy with antibiotics within the last 2 weeks before the start of the study and/or throughout the entire course of the study, systemic therapy with immuno-suppressive drugs (e.g., corticosteroids) and/or antihistamines (e.g., antiallergics) within the last 30 days before the start of the study; supporting therapy against atopic dermatitis (UV therapy, probiotic homeopathy, etc.) within the last 2 weeks before the start of the study; antiseptic or antibacterial wash or topical products within 4 weeks before the start of the study; and topical corticosteroids at the test area within the last 2 weeks before the start of the study. The subjects were not allowed to use any detergents or leave cosmetics on the bend of arms at least 3 days before sample collection.

On the sample collection day, the dermatologist clarified and discussed the AD symptoms with the patients, and then the severity of AD was evaluated based on the local SCORAD index specifically in the bend of the arms, not the whole area of the body. Six parameters, including erythema, edema and papules, oozing and crusts, excoriation, lichenification, and xerosis of AD patients, were evaluated by the dermatologist according to the following scale: 0 = absent, 1 = slight, 2 = moderate, 3 = strong. The local SCORAD per test area was the sum of all scores for all six abovementioned parameters (minimum = 0, maximum = 18). The subjects with the sum of score of at least 4 in either one of the bends of the arms were recruited in this study. The local SCORAD information of the bend of the arms of each AD patient is listed in Table S1.

Two skin swab samples were collected from the lesion and non-lesion (5 cm away from the lesion) sites in AD patients by a trained physician. Two swabs were also collected in the bend of the arm in healthy subjects. One swab was used for 16S rRNA gene sequencing analysis; another one was used for metabolomics analysis.

For skin microbiome analysis, an area of 5 cm in diameter was sampled by swabbing the skin for 30 s with a sterile flock swab, which was dipped into an aliquot of phosphate-buffered saline (PBS). The lateral edge of it and the swab were rubbed across the entire defined area while being rotated between the thumb and forefinger for 30 s. More specifically, the rotating swab was rubbed back and forth in a crosswise manner in the defined area in the same fashion for each subject to maintain consistency. After 30 s, the head of each swab was placed into a sterile microcentrifuge tube and aseptically cut from the breakpoint of the handle before closing the tube lid. All the samples were frozen at −80°C until further analysis. A blank swab, which was dipped in PBS without taking any skin samples, was stored in a sterile microcentrifuge tube for negative control.

For metabolomics analysis, an area of 5 cm in diameter was sampled with a pre-cleaned Puritan cotton swab, which was dipped into an aliquot of ethanol-water solvent (1:1 vol/vol) mix shortly before sampling. The swabbing procedure was the same as the one for microbiome sampling. All the samples were frozen at −80°C until further analysis.

### 16S rRNA gene sequencing and data analysis

The v1-3 region of 16S rRNA gene sequencing was conducted by RTL Genomics (Lubbock, TX, USA) as previously described (42). The v1-3 hypervariable regions of the 16S rRNA genes were sequenced on the MiSeq platform (Illumina, Inc., San Diego, CA, USA). DNA was extracted via KingFisher FLEX instrument and using Zymo ZR-96 magbead kit following the manufacturer’s instructions. The extraction protocol was modified to include a mechanical lysis step with a Qiagen TissueLyser.

Samples were amplified for sequencing in a two-step process. The forward primer was constructed (5′−3′) with the forward Illumina overhang adapter (TCGTCGGCAGCGTCAGATGTGTATAAGAGACAG) added to the 28F primer (5′ GAG TTT GAT CNT GGC TCA G 3′). The reverse primer was constructed (5′−3′) with the reverse Illumina overhang adapter (GTCTCGTGGGCTCGGAGATGTGTATAAGAGACAG) added to the 519R primer (5′ GTN TTA CNG CGG CKG CTG 3′). Amplifications were performed in 25-µL reactions with Qiagen HotStar Taq master mix (Qiagen Inc., Valencia, CA, USA), 1 µL of each 5 µM primer, and 1 µL of template. Reactions were performed on ABI Veriti thermocyclers (Applied Biosystems, Carlsbad, CA, USA) under the following thermal profile: 95°C for 5 min, then 10 cycles of 94°C for 30 s, 50°C for 90 s (+0.5°C per cycle), 72°C for 1 min, followed by 25 cycles of 94°C for 30 s, 54°C for 90 s, 72°C for 1 min, and finally, one cycle of 72°C for 10 min and 4°C hold.

Products from the first-stage amplification were added to a second PCR based on qualitatively determined concentrations. Primers for the second PCR were designed based on the Illumina Nextera PCR primers as follows: Forward, AATGATACGGCGACCACCGAGATCTACAC[i5index]TCGTCGGCAGCGTC, and Reverse, CAAGCAGAAGACGGCATACGAGAT[i7index]GTCTCGTGGGCTCGG. The second-stage amplification was run with the following thermal profile: 95°C for 5 min, then 10 cycles of 94°C for 30 s, 54°C for 40 s, 72°C for 1 min, followed by one cycle of 72°C for 10 min and 4°C hold.

Amplification products were visualized with eGels (Life Technologies). Products were then pooled equimolar by band intensity, and each pool was size selected in two rounds using SPRIselect beads (BeckmanCoulter) in a 0.75 ratio for both rounds. Size-selected pools were then quantified using Qubit 4 fluorometer (Life Technologies) and loaded on an Illumina MiSeq 2 × 300 flow cell at 10 pM for sequencing. Additionally, negative controls were introduced starting from sample collection (swabs dipped in PBS at time of collection), extraction, and from PCR preparation, which were otherwise treated identically to samples and sequenced alongside study samples.

Bioinformatic processing and quality filtering generally followed previous work (43). Denoising of sequence reads chimera detection and stitching of 2 × 300 paired reads were conducted using Usearch7 (44), UCHIME (45), and PEAR (46), respectively. Quality-filtered and assembled reads were clustered into OTUs at 97% sequence similarity threshold using the UPARSE algorithm. OTU assignment was then completed using USEARCH global search as described by Bokulich (47) compared to the RTL Genomics in-house taxonomic reference database, which is adapted from the National Center for Biotechnology Information reference database with additional curation. Multiple sequence alignment and phylogenetic tree estimation of representative OTU sequences were performed for downstream analysis using MUSCLE (48) and FastTree2 (49).

Statistical analysis was conducted using an R programming environment. Prior to analysis, OTU-level filtering was conducted to exclude OTUs classified as “no hits” or Ralstonia (a common reagent contaminant). The negative controls garnered very few reads, ranging from 0 to 20 reads across 13 negative controls, and contamination was considered negligible. A read count normalization to 9,600 reads was also implemented using scaling with ranked subsampling (50). Bacterial alpha and beta diversities were summarized by Shannon diversity and weighted UniFrac dissimilarity (51). The alpha and beta diversity differences between ADA lesion, AD nonlesion, and the healthy group were analyzed using ANOVA and PERMANOVA, as implemented in the R function ADONIS. Microshades were used for the visualization of microbial abundance (52). PCoA was used to visualize compositional similarity among microbiome communities. To evaluate differences in the relative abundances of taxa between cohorts, the ANCOM-BC (53) procedure was carried out on taxa present in at least 20% of the samples, using the arguments struc zero = T and neg lb = T. Holm’s method was used to adjust *P* values and account for multiple test correction. *P* < 0.05 is considered statistically significant. A focused analysis of *S. aureus* and *S. epidermidis* group differences was conducted on both the relative abundance and arcsine-transformed relative abundance.

### Sample preparation, metabolomics, and data processing

The cotton buds of all samples (30 healthy, 30 ADNL, and 30 ADL—a total of 90 samples) were added into a 96-deep well plate (2 mL). Using a multichannel pipette, aliquots of 500 µL of ethanol/H_2_O (1:1) were added to each well for extraction, containing 1 mM of sulfadimethoxine (internal standard to extraction). The plates were sonicated for 5 min in an ultrasound bath, Branson 2800 (Danbury, CT, USA), vortexed (~10 s), and then, the swabs were removed with tweezers, and the extracts were dried by speed vacuum. Finally, they were stored at −80°C until resuspension.

The samples were resuspended with 200 µL of ACN/H_2_O (1:1) containing 1 mM of sulfachloropyridazine (internal standard to metabolomic analyses), and then, the plates were sonicated for 5 min in an ultrasound bath, Branson 2800 (Danbury, CT, USA), vortexed (~10 s), and centrifuged for 20 min at 2,000 rpm and 4°C. Thus, using a multichannel pipette, aliquots of 150 µL of each sample were transferred to a 200-µL ThermoScientific 96-well plate for LC-MS/MS analysis. A mix of ACN:H_2_O (1:1) containing 1 mM of sulfachloropyridazine was used as a blank, and a mix of sulfamethazine (C_12_H_14_N_4_O_2_S), sulfamethizole (C_9_H_10_N_4_O_2_S_2_), sulfachloropyridazine (C_10_H_9_ClN_4_O_2_S), sulfadimethoxine (C_12_H_14_N_4_O_4_S), amitriptyline (C_20_H_23_N·HCl), and coumarin-314 (C_18_H_19_NO_4_) was used as quality control (QC).

The metabolomic analyses were performed in a Vanquish UHPLC system coupled to a Q-Exactive orbitrap mass spectrometer (Thermo Fisher Scientific, Waltham, MA, USA), controlled by Thermo SII for Xcalibur software (Thermo Fisher Scientific, Waltham, MA, USA). The chromatographic analysis was carried out on a Kinetex C18 column (50 × 2.1-mm, 1.7-µm particle size, 100-A pore size, Phenomenex, Torrance, CA, USA). A high-pressure binary system was used for gradient elution. The column and autosampler were kept at 40°C, and 25°C, respectively. The flow rate was 0.5 mL/min, and the elution was carried out using ultra-pure water (solvent A) and acetonitrile (solvent B), both acidified with 0.1% of formic acid (FA). The gradient method was set as follows: 0–0.5 min, 5% B; 0.5–8.0 min, 5%–100% B; 8.0–11.0 min, 100% B; 11.0–12.0 min, 100%-5% B; and finally, 12.0–14.0 min 5% B to stabilize the system before the subsequent analysis.

For mass spectrometry analyses, data-dependent acquisition (DDA) was used in an *m/z* range from 80 to 2,000 Da with an electrospray source operating in the positive ionization mode. Before data acquisition, sodium formate solution (Thermo Fisher Scientific San Diego, CA, USA) was used for external calibration with an error rate of less than 0.5 ppm. The spray voltage was set to 3.5 kV, sheath N_2_ gas pressure was set to 35 psi, and auxiliary N_2_ gas pressure was set to 10 psi. The ionization source was kept at 270°C, a 60 V S-lens RF level was applied, and the auxiliary gas heater was kept at 440°C. Full-scan MS^1^ was performed at 1.0 × 10^6^ with a resolution of 35,000 and a maximum ion injection of 100 ms. MS^2^ experiments were performed with a resolution of 17,500 with a max IT time of 60 ms, and topK6 was used for the six most abundant precursor ions per MS^1^. The MS^2^ precursor isolation window was set to 2 Da with an offset of 0.5 Da. The normalized collision energy was set to a ramp from 20 to 40 eV, and the exclusion (MS^1^ and MS^2^) for unassigned ion charge states was set to 5 S-Lens, as well as isotope peaks.

The LC-MS/MS data were converted from RAW standard data format (Thermo Fisher Scientific, Waltham, MA. USA) to mzML format using MSConvert 3.0.2 (54). Thereafter, the data were uploaded to the MassIVE repository (data set MSV000090788) (55). The LC-MS/MS data were processed using MZmine 3.1.0 (56). The mass detection of MS^1^ and MS^2^ levels was performed using a signal noise of 1.0 × 10^5^ and 5.0 × 10^3^, respectively. The ADAP chromatogram builder was used to build the chromatogram, and a minimum group size of scans was set to 3, the minimum intensity of the group to 1.0 × 10^5^, and the highest to 3.0 × 10^5^ with an *m/z* tolerance of 10 ppm. The ADAP resolver module (wavelets) was applied to chromatographic deconvolution. Then, intensity window S/N was used as an S/N estimator with a signal-to-noise ratio set to 10, a minimum feature height of 1.0 × 10^4^, a coefficient of peak area of 1.70, a peak duration from 0.05 to 2.0 min, and an RT wavelet range used to build a matrix of coefficients from 0.05 to 0.10 min. The isotope peak grouper module was applied to detect the isotopes with an *m/z* and RT tolerance of 10.0 ppm, and 0.2 min (charge 1 was used as standard) for the most intense isotope. To remove the duplicate features and aligner, the same *m/z* and RT tolerances were used, and the weight for *m/z* and RT was set to 3:1, respectively. The resulting peak list was then filtered to remove features from the blanks, and features with isotope patterns and MS^2^ spectra associated were kept. Thus, a filtered peak list containing 9,541 features was exported as a .mgf file and a .csv file containing feature information, which was used for downstream statistical analysis.

The outputs containing MS features were then used in the Feature-Based Molecular Networking workflow (23) on the GNPS platform (https://gnps.ucsd.edu/). The job can be found at the following link (https://gnps.ucsd.edu/ProteoSAFe/status.jsp?task=a36305db40414b6ea9c5b9a5de380bea). The parameters used were set as follows: the tolerances for the precursor ion mass (MS^1^) and the MS^2^ fragment ion were set to 0.02 and 0.02 Da, respectively. A cosine of 0.6 and a minimum of 4 MS^2^ matches were applied to create the molecular network. The maximum number of neighbor nodes for each node was set to 10 for most similar nodes, and the maximum number of molecular families was set at 100. All spectra contained in the molecular networks were compared to the reference spectra available in the GNPS spectral libraries (57), and a cosine of 0.6 and a minimum of 3 MS^2^ matches were applied. The *in silico* suspect library (25) was also used. The molecular network file was visualized using Cytoscape 3.9.1 (58).

Statistical analyses were performed in Python using Jupyter Notebook. Random forest was performed using the Random Forest Classifier Scikit-Learn package. The Kruskal–Wallis test followed by the Wilcoxon signed-rank test were used to assess the differences in samples. Multivariate analysis was performed using the “pandas,” “sklearn.decomposition.PCA,” and “sklearn.cross_decomposition.PLSRegression” packages. The methods detailed are available in the supplemental material.

### Microbiome and metabolomic correlation analysis

Co-occurrence probabilities between microbes and molecules were calculated using the mmvec tool (26). This analysis was performed using mmvec 1.0.4 (https://github.com/biocore/mmvec) as a Qiime2 plugin (59). As inputs for this analysis, a relative abundance matrix for the sequencing data should be provided along with a feature abundance table for the ion features. mmvec then uses a neural networking approach to calculate the conditional probabilities of observing molecules based on the abundance of each microbe. In this study, four mmvec analyses were conducted: (i) 90 samples comprising the three groups (healthy, atopic dermatitis with the lesion, and atopic dermatitis without lesion); ii) 60 samples comprising the healthy and atopic dermatitis without lesion groups; (iii) 60 samples comprising the healthy and atopic dermatitis with lesion groups; and (iv) 60 samples comprising atopic dermatitis with and without the lesion groups. For the analyses conducted with 60 samples, the microbial and molecule features that were only detected in the excluded group were removed. The mmvec parameters were as follows: –p-learning-rate 1e-3, –p-epochs 300. All other parameters for the analyses were set as the default values. Emperor (20) was used to inspect the feature–feature biplots visually. The spheres were colored based on which group the molecules were most abundant, and the arrows indicate the 30 most important OTUs retrieved from the analyses (“importance” was calculated for each feature based on the magnitude of the vector using Euclidean distance from the origin). Tables of co-occurrence values for each microbe with each molecule were also generated for all four analyses. Emperor (60) was used to inspect the feature–feature biplots visually. These co-occurrence probabilities were also visualized as networks using Cytoscape 3.9.1 (58).

## Data Availability

The 16S rRNA sequencing data are publicly available at https://www.ncbi.nlm.nih.gov/bioproject/998761. The raw data of metabolomics are publicly available online at MassIVE (https://massive.ucsd.edu/) under the accession number MSV000090788. All data generated or analyzed during this study are included in this published article (and its supplemental material).

## SUPPLEMENTAL MATERIAL

Supplemental Material msystems.01119-23-s0001.docx Supplemental figures and tables.

ASM does not own the copyrights to Supplemental Material that may be linked to, or accessed through, an article. The authors have granted ASM a non-exclusive, world-wide license to publish the Supplemental Material files. Please contact the corresponding author directly for reuse.
