# Supplementary material for: Co-occurrence network analysis reveals the alterations of the skin microbiome and metabolome in adults with mild to moderate atopic dermatitis
Source: mSystems. 2024 Feb 6;9(3):e01119-23. doi: 10.1128/msystems.01119-23 (PMC10949451; doi:10.1128/msystems.01119-23)
Supplement: Supplemental Material — Supplemental figures and tables. [file msystems.01119-23-s0001.docx]

**Supplementary Materials**

**
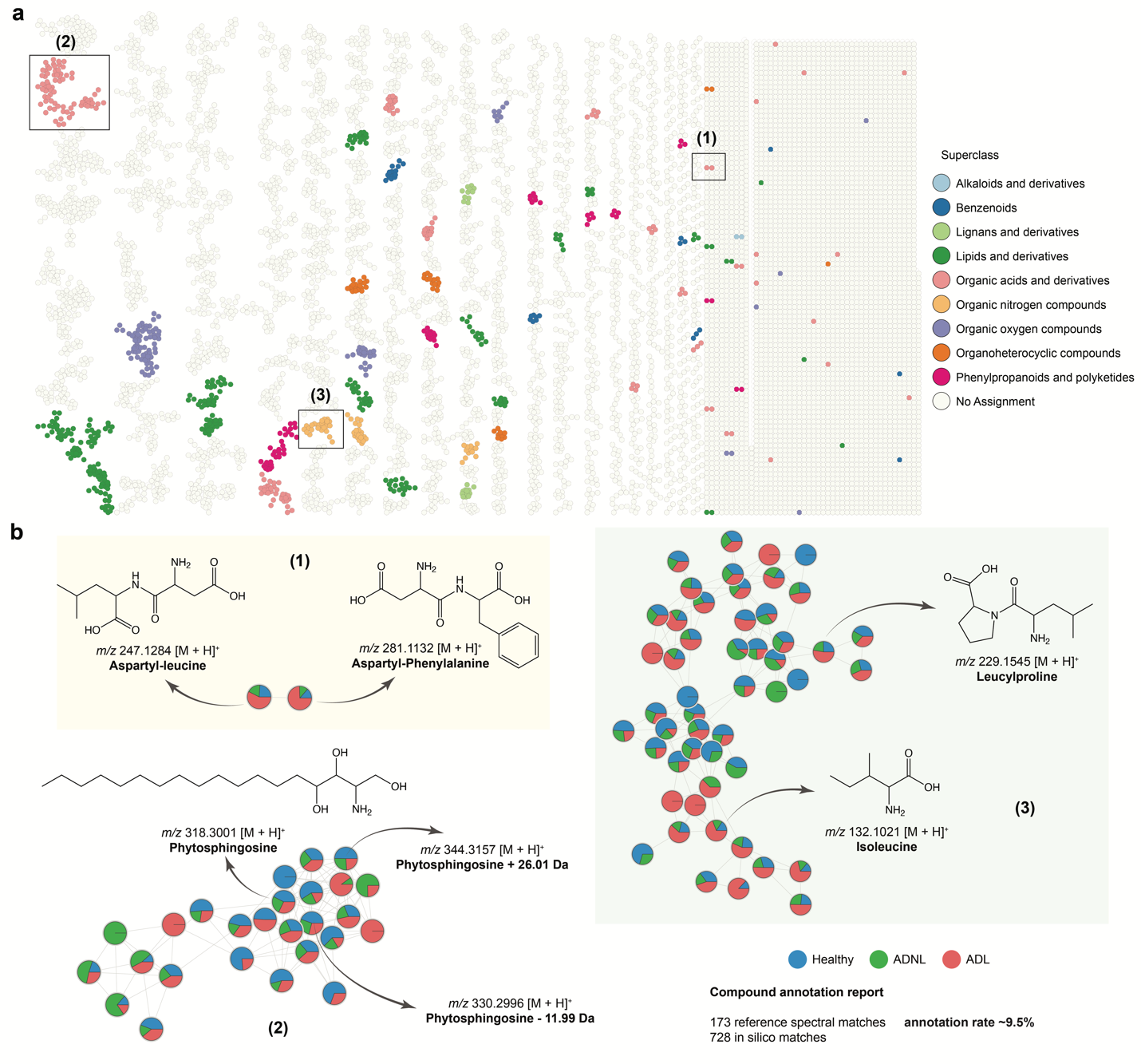
**

**FIG S1. Molecular network and examples of annotated molecules.** a) Chemical classification retrieved from MolNetEnhancer outcomes. Overall, 901 features (out of 9,541) had matches against the GNPS spectral libraries (annotation score ~9,5%). Among them, 173 are against reference MS/MS spectra, and 728 are related as *in silico* annotation from the suspect library (1). b) Examples of annotated molecules among those classified by Random Forest (top 15).


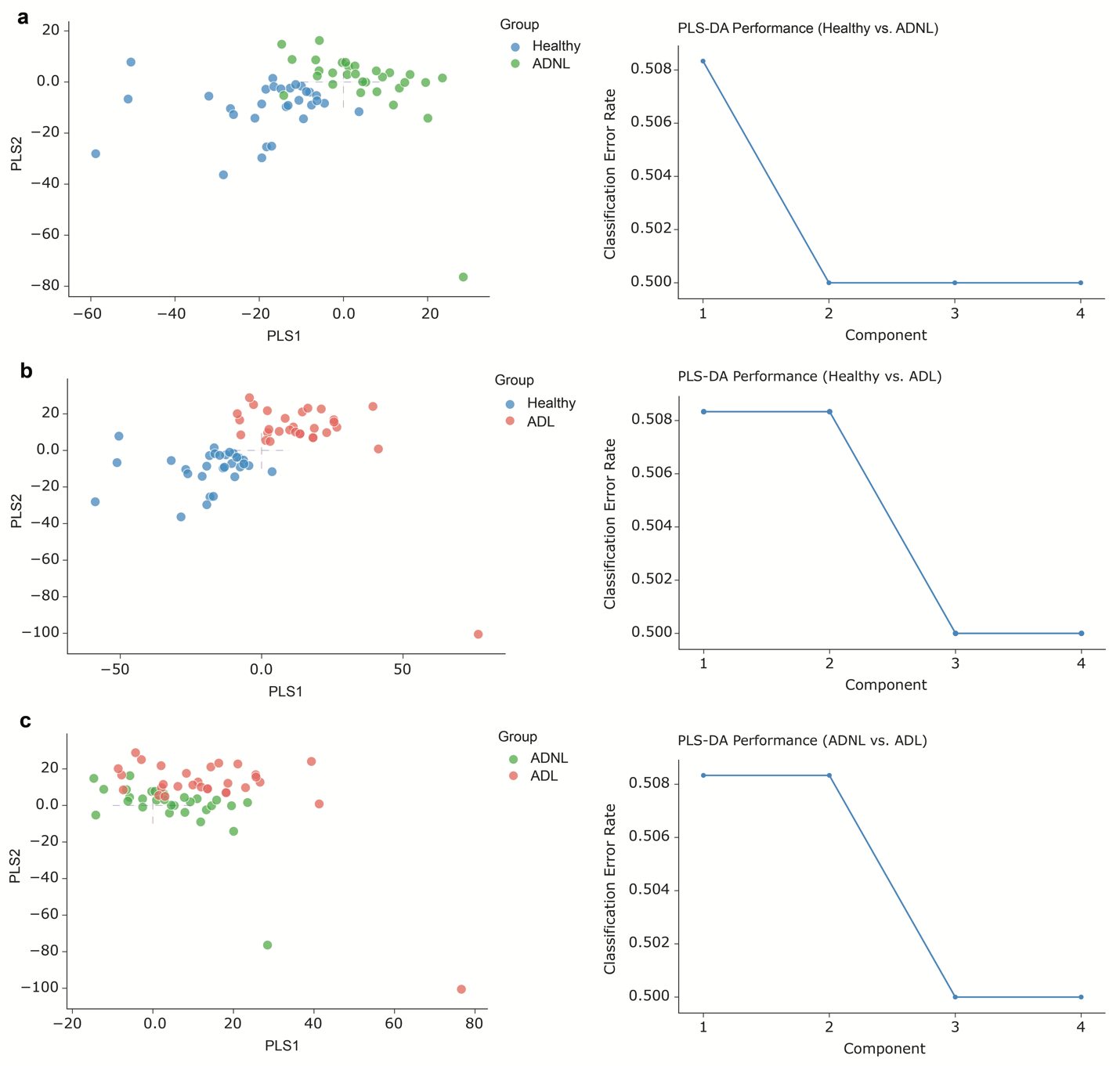


**FIG S2. Different groups lead to distinct skin biochemical profiles.** Pairwise PLS-DA models show close to perfect separation between healthy and unhealthy groups (a, b). PLS-DA plot of ADNL vs. ADL (c) reveals possible misclassification. Model performances were calculated using random 4-fold cross-validation and are illustrated to each PLS-DA model.


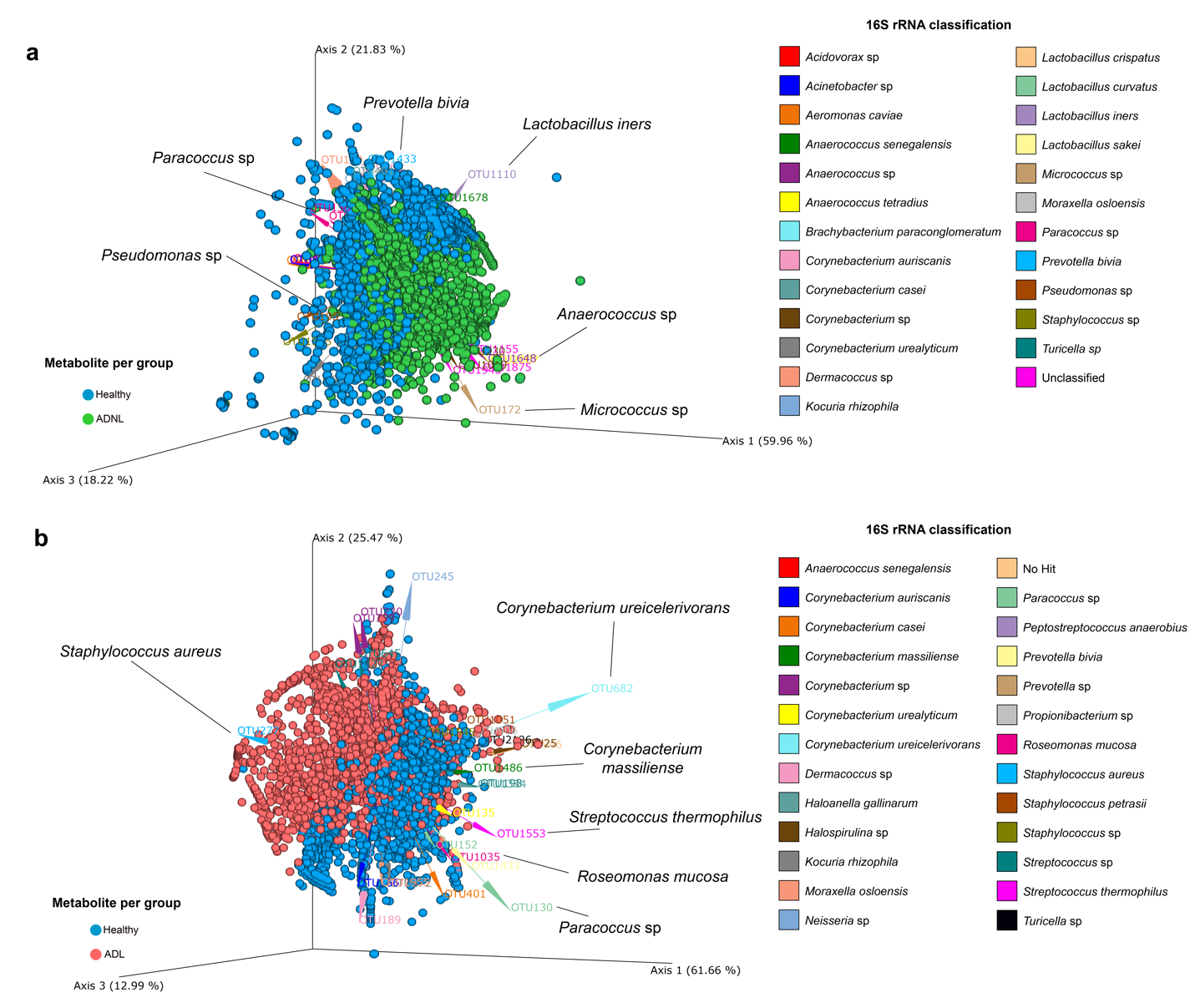


**FIG S3. Microbe-molecule co-occurrence biplots**. The mmvec per pairs analyses were performed including samples from the healthy, atopic dermatitis without lesions and atopic dermatitis with lesion groups (90 samples). (a) the healthy and atopic dermatitis without lesion groups (60 samples), and (b) the healthy and atopic dermatitis with lesion groups (60 samples). Spheres in the biplot represent the molecules, while the arrows represent the microbes. Spheres were colored based on which group each molecule was most abundant in, while microbes were colored based on which species they belonged to. Small angles between arrows indicate microbes co-occurring with each other. Similarly, closer spheres indicate molecules co-occurring. Arrows pointing towards a group of molecules indicate microbe-molecule co-occurrence. This biplot shows the 30 most important OTUs (higher vector magnitude).


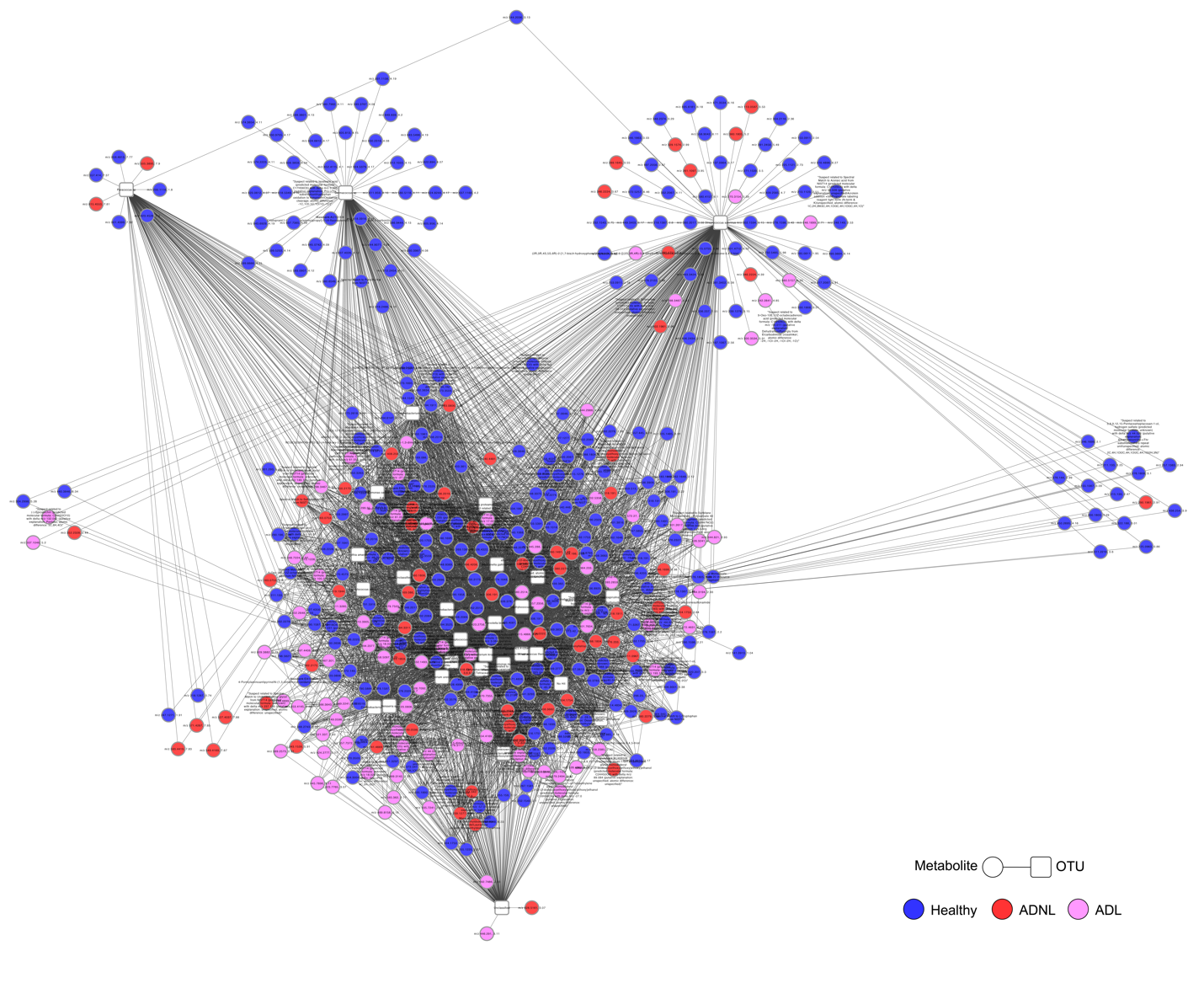


**FIG S4. The network was obtained from the mmvec co-occurrences probabilities for all the samples in the study (90 samples, three groups).** Squares indicate the top 30 OTUs and circles indicate molecules, while edges between them indicate co-occurrences probabilities (>5.0). The OTUs and molecule features are labeled (for molecules with spectral annotation, it is specified; features without annotation are shown as *m/z* values in addition to their retention time). Nodes are relative to the molecules and are colored based on which group each molecule was most abundant (blue: healthy; red: atopic dermatitis without lesion; pink: atopic dermatitis with lesion).


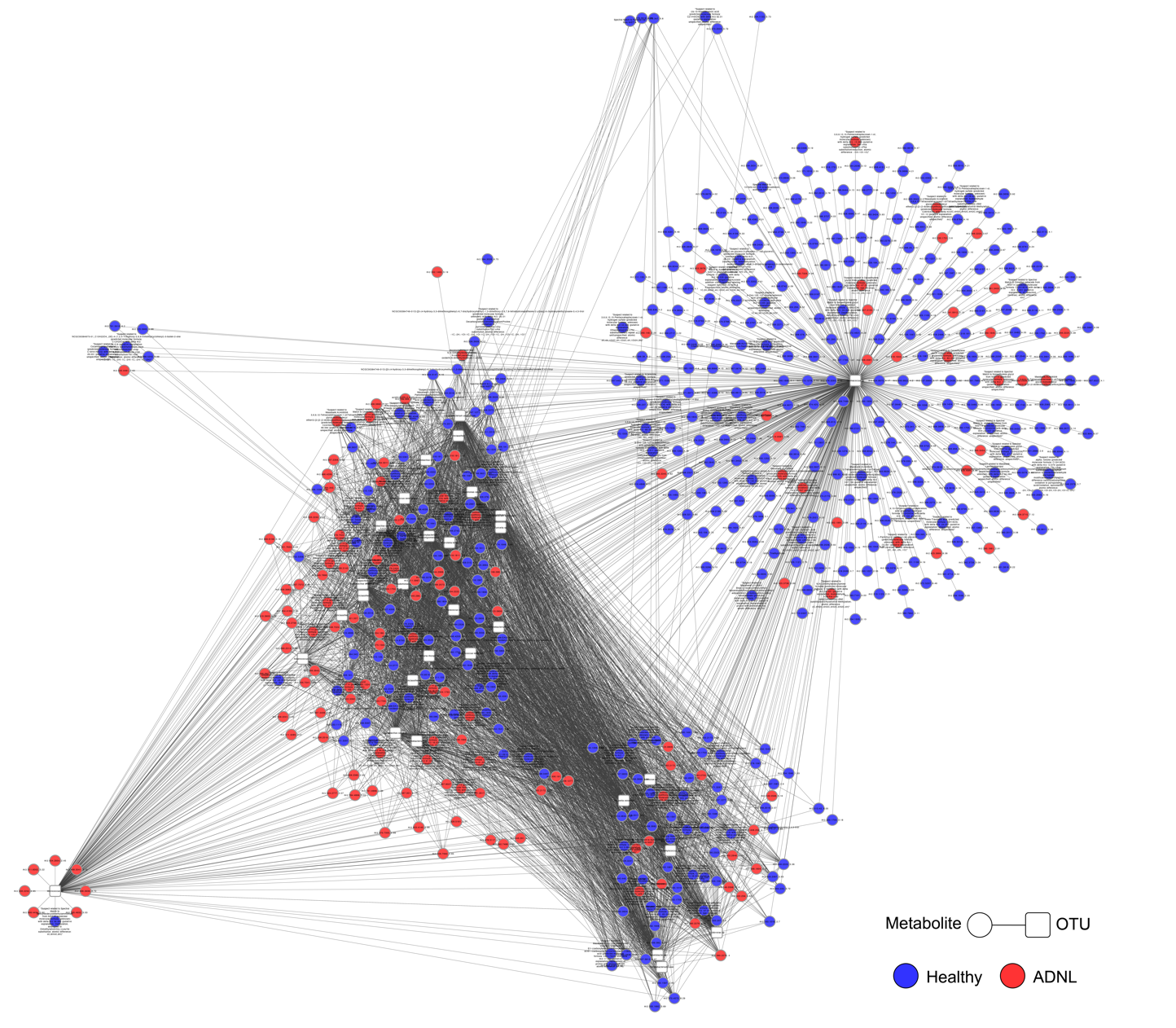


**FIG S5. The network was obtained from the mmvec co-occurrences probabilities for the samples from the healthy and atopic dermatitis without lesion groups (60 samples).** Squares indicate the top 30 OTUs and circles indicate molecules, while edges between them indicate co-occurrences probabilities (>5.0). The OTUs and molecule features are labeled (for molecules with spectral annotation, it is specified; features without annotation are shown as *m/z* values in addition to their retention time). Nodes are relative to the molecules and are colored based on which group each molecule was most abundant (blue: healthy; red: atopic dermatitis without lesion).


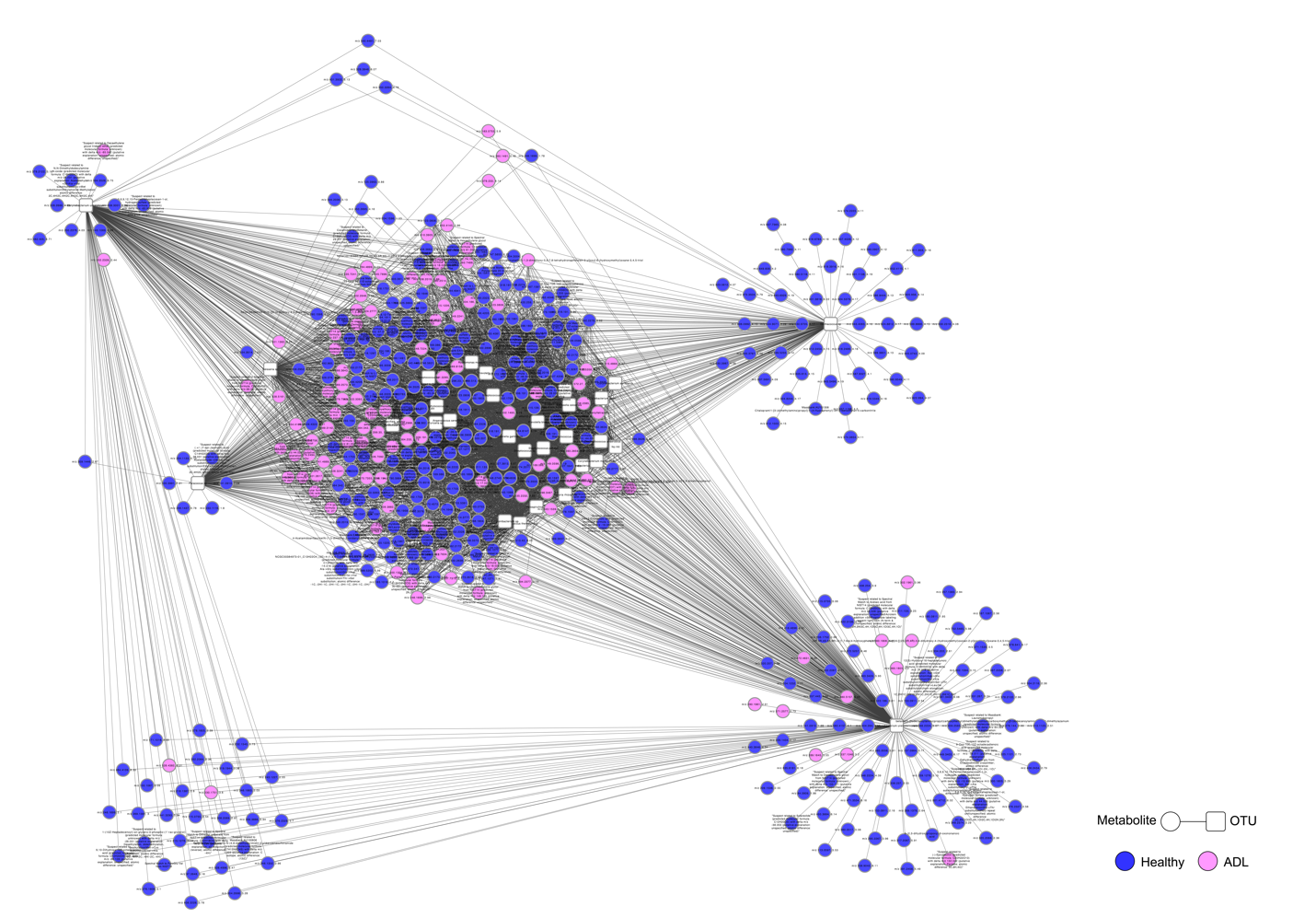


**FIG S6. The network was obtained from the mmvec co-occurrences probabilities for the samples from the healthy and atopic dermatitis with lesion groups (60 samples).** Squares indicate the top 30 OTUs and circles indicate molecules, while edges between them indicate co-occurrences probabilities (>5.0). The OTUs and molecule features are labeled (for molecules with spectral annotation, it is specified; features without annotation are shown as *m/z* values in addition to their retention time). Nodes are relative to the molecules and are colored based on which group each molecule was most abundant (blue: healthy; pink: atopic dermatitis with lesion).

**Table S1. Scores for local SCORAD.** Subjects with Atopic Dermatitis evaluated by a dermatologist according to the following scale: 0 = Absent, 1 = Slight, 2 = Moderate, 3 = Strong.

| **Subject No.** | **Age (yr)** | **Xerosis** | | **Erythema (SCORAD)** | | **Edema and Papules** | | **Oozing and Crusts** | | **Excoriation** | | **Lichenification** | | **SUM of the 6 parameters** | **SUM of the 6 parameters** |
| --- | --- | --- | --- | --- | --- | --- | --- | --- | --- | --- | --- | --- | --- | --- | --- |
|  |  | **L** | **R** | **L** | **R** | **L** | **R** | **L** | **R** | **L** | **R** | **L** | **R** | **L** | **R** |
| 1 | 29 | 1 | 2 | 1 | 1 | 0 | 0 | 0 | 0 | 1 | 1 | 2 | 1 | 5 | 5 |
| 4 | 53 | . | 2 | . | 2 | . | 0 | . | 0 | . | 0 | . | 2 | . | 6 |
| 5 | 44 | 2 | 2 | 1 | 2 | 0 | 0 | 0 | 0 | 0 | 0 | 2 | 2 | 5 | 6 |
| 8 | 58 | . | 2 | . | 2 | . | 0 | . | 0 | . | 0 | . | 1 | . | 5 |
| 9 | 28 | 1 | 1 | 1 | 1 | 0 | 0 | 0 | 1 | 1 | 2 | 1 | 1 | 4 | 6 |
| 15 | 24 | 1 | 1 | 3 | 2 | 1 | 1 | 0 | 0 | 0 | 0 | 1 | 1 | 6 | 5 |
| 18 | 21 | 2 | 2 | 1 | 1 | 0 | 0 | 0 | 0 | 0 | 0 | 1 | 1 | 4 | 4 |
| 24 | 31 | 2 | 2 | 2 | 2 | 0 | 0 | 0 | 0 | 1 | 1 | 1 | 1 | 6 | 6 |
| 32 | 52 | 2 | 2 | 1 | 1 | 0 | 0 | 0 | 0 | 0 | 0 | 1 | 1 | 4 | 4 |
| 36 | 19 | 2 | 2 | 3 | 3 | 0 | 0 | 0 | 0 | 1 | 1 | 2 | 2 | 8 | 8 |
| 41 | 27 | 1 | 1 | 2 | 2 | 0 | 0 | 0 | 0 | 0 | 0 | 1 | 1 | 4 | 4 |
| 42 | 24 | 2 | 2 | 1 | 1 | 0 | 0 | 0 | 0 | 1 | 1 | 1 | 1 | 5 | 5 |
| 43 | 37 | . | 2 | . | 2 | . | 0 | . | 0 | . | 0 | . | 1 | . | 5 |
| 44 | 29 | 2 | 2 | 1 | 1 | 0 | 0 | 0 | 0 | 0 | 0 | 1 | 1 | 4 | 4 |
| 45 | 38 | 2 | 2 | 1 | 1 | 1 | 1 | 0 | 0 | 0 | 0 | 1 | 1 | 5 | 5 |
| 46 | 21 | 2 | 2 | 3 | 2 | 3 | 2 | 0 | 0 | 0 | 0 | 1 | 1 | 9 | 7 |
| 47 | 21 | . | 2 | . | 2 | . | 1 | . | 0 | . | 1 | . | 2 | . | 8 |
| 48 | 33 | 3 | 3 | 3 | 3 | 2 | 3 | 2 | 2 | 3 | 2 | 3 | 3 | 16 | 16 |
| 49 | 34 | 2 | 2 | 2 | 2 | 1 | 1 | 1 | 1 | 1 | 1 | 3 | 3 | 10 | 10 |
| 50 | 71 | 3 | 3 | 3 | 3 | 3 | 3 | 0 | 0 | 1 | 1 | 3 | 3 | 13 | 13 |
| 51 | 61 | 3 | 3 | 1 | 1 | 2 | 2 | 0 | 0 | 0 | 0 | 3 | 3 | 9 | 9 |
| 52 | 21 | 2 | 2 | 1 | 1 | 0 | 0 | 0 | 0 | 0 | 0 | 2 | 2 | 5 | 5 |
| 53 | 18 | 2 | 2 | 1 | 1 | 2 | 2 | 1 | 0 | 1 | 1 | 2 | 2 | 9 | 8 |
| 54 | 36 | 3 | 3 | 2 | 2 | 2 | 1 | 1 | 1 | 2 | 1 | 3 | 3 | 13 | 11 |
| 55 | 28 | 3 | 3 | 2 | 1 | 0 | 0 | 0 | 0 | 0 | 0 | 1 | 1 | 6 | 5 |
| 56 | 45 | . | 1 | . | 3 | . | 0 | . | 0 | . | 0 | . | 2 | . | 6 |
| 57 | 38 | 3 | 3 | 1 | 2 | 0 | 1 | 0 | 0 | 0 | 0 | 2 | 2 | 6 | 8 |
| 58 | 32 | 2 | 2 | 1 | 0 | 1 | 0 | 1 | 0 | 1 | 0 | 2 | 2 | 8 | 4 |
| 59 | 32 | 2 | 2 | 1 | 1 | 0 | 0 | 0 | 0 | 0 | 0 | 1 | 1 | 4 | 4 |
| 60 | 69 | 2 | 2 | 1 | 1 | 0 | 0 | 0 | 0 | 0 | 0 | 1 | 1 | 4 | 4 |

Note: L = Left; R = Right.

**Table S2. Species Identified by ANCOMBC vary between the groups.** The “q_val” is the bias-corrected p-value and values under ADL, ADNL, and Healthy are mean relative abundance for species found to be differentially abundant.

| **OTU** | **Beta** | **q_val** | **STD** | **W** | **ADL** | **ADNL** | **Healthy** |
| --- | --- | --- | --- | --- | --- | --- | --- |
| *S. aureus* | -3.6598601 | 0.0000166 | 0.6832911 | -5.356223 | 0.18642 | 0.06614 | 0.00129 |
| *S. epidermidis* | -1.5169886 | 0.0016923 | 0.3411558 | -4.446615 | 0.16700 | 0.10800 | 0.03740 |
| Unclassified | 1.3657898 | 0.0049949 | 0.3246484 | 4.206982 | 0.00230 | 0.00564 | 0.00916 |
| *Brevundimonas* sp | 1.5025050 | 0.0066782 | 0.3629539 | 4.139658 | 0.00125 | 0.00381 | 0.00572 |
| *Pseudomonas stutzeri* | 1.2499271 | 0.0111228 | 0.3109386 | 4.019852 | 0.00184 | 0.00363 | 0.00491 |
| *Sphingomonas* sp | 0.9923095 | 0.0175695 | 0.2538173 | 3.909543 | 0.00032 | 0.00073 | 0.00173 |

Note: OTU: operational taxonomic unit; Beta: coefficient obtained from the ANCOM-BC log-linear model (the unstandardized effect size); q_val: adjusted p-values using the default “holm” method; STD: the standard errors of the beta value; W: the test statistic calculated by W = beta/se (the standardized effect size)

**Table S3. Top 30 OTUs highlighted in the biplots obtained from mmvec analysis.** All groups (Healthy, ADNL, and ADL) were used to extract the OTUs.

| **OTU** | **phylum** | **class** | **order** | **family** | **genus** | **species** |
| --- | --- | --- | --- | --- | --- | --- |
| OTU1006 | Actinobacteria | Actinobacteria | Micrococcales | Micrococcaceae | *Rothia* | *Rothia amarae* |
| OTU1019 | Actinobacteria | Actinobacteria | Corynebacteriales | Corynebacteriaceae | *Corynebacterium* | *Corynebacterium* sp |
| OTU1035 | Proteobacteria | Alphaproteobacteria | Rhodospirillales | Acetobacteraceae | *Roseomonas* | *Roseomonas mucosa* |
| OTU1260 | Actinobacteria | Actinobacteria | Corynebacteriales | Corynebacteriaceae | *Corynebacterium* | *Corynebacterium bovis* |
| OTU1282 | Firmicutes | Bacilli | Lactobacillales | Streptococcaceae | *Streptococcus* | *Streptococcus sp* |
| OTU130 | Proteobacteria | Alphaproteobacteria | Rhodobacterales | Rhodobacteraceae | *Paracoccus* | *Paracoccus sp* |
| OTU135 | Actinobacteria | Actinobacteria | Corynebacteriales | Corynebacteriaceae | *Corynebacterium* | *Corynebacterium urealyticum* |
| OTU1384 | Bacteroidetes | Flavobacteriia | Flavobacteriales | Flavobacteriaceae | *Haloanella* | *Haloanella gallinarum* |
| OTU1433 | Bacteroidetes | Bacteroidia | Bacteroidales | Prevotellaceae | *Prevotella* | *Prevotella bivia* |
| OTU1545 | Firmicutes | Bacilli | Bacillales | Staphylococcaceae | *Staphylococcus* | *Staphylococcus* sp |
| OTU1553 | Firmicutes | Bacilli | Lactobacillales | Streptococcaceae | *Streptococcus* | *Streptococcus thermophilus* |
| OTU1699 | Actinobacteria | Actinobacteria | Corynebacteriales | Corynebacteriaceae | *Corynebacterium* | *Corynebacterium kroppenstedtii* |
| OTU173 | Actinobacteria | Actinobacteria | Corynebacteriales | Corynebacteriaceae | *Corynebacterium* | *Corynebacterium* sp |
| OTU1875 | Firmicutes | Unclassified | Unclassified | Unclassified | Unclassified | Unclassified |
| OTU189 | Actinobacteria | Actinobacteria | Micrococcales | Dermacoccaceae | *Dermacoccus* | *Dermacoccus sp* |
| OTU1924 | Proteobacteria | Gammaproteobacteria | Enterobacteriales | Enterobacteriaceae | *Serratia* | *Serratia proteamaculans* |
| OTU195 | Proteobacteria | Gammaproteobacteria | Pseudomonadales | Pseudomonadaceae | *Pseudomonas* | *Pseudomonas sp* |
| OTU2062 | Actinobacteria | Actinobacteria | Corynebacteriales | Corynebacteriaceae | *Corynebacterium* | *Corynebacterium genitalium* |
| OTU228 | Proteobacteria | Gammaproteobacteria | Aeromonadales | Aeromonadaceae | *Aeromonas* | *Aeromonas caviae* |
| OTU230 | Actinobacteria | Actinobacteria | Corynebacteriales | Corynebacteriaceae | *Corynebacterium* | *Corynebacterium* sp |
| OTU245 | Proteobacteria | Betaproteobacteria | Neisseriales | Neisseriaceae | *Neisseria* | *Neisseria sp* |
| OTU25 | Cyanobacteria | Unclassified | Oscillatoriales | Unclassified | *Halospirulina* | *Halospirulina sp* |
| OTU277 | Firmicutes | Bacilli | Bacillales | Staphylococcaceae | *Staphylococcus* | *Staphylococcus aureus* |
| OTU401 | Actinobacteria | Actinobacteria | Corynebacteriales | Corynebacteriaceae | *Corynebacterium* | *Corynebacterium casei* |
| OTU566 | Actinobacteria | Actinobacteria | Corynebacteriales | Corynebacteriaceae | *Corynebacterium* | *Corynebacterium auriscanis* |
| OTU682 | Actinobacteria | Actinobacteria | Corynebacteriales | Corynebacteriaceae | *Corynebacterium* | *Corynebacterium ureicelerivorans* |
| OTU716 | Firmicutes | Unclassified | Unclassified | Unclassified | Unclassified | Unclassified |
| OTU759 | Actinobacteria | Actinobacteria | Corynebacteriales | Corynebacteriaceae | *Corynebacterium* | *Corynebacterium* sp |
| OTU833 | Firmicutes | Bacilli | Lactobacillales | Streptococcaceae | *Streptococcus* | *Streptococcus sobrinus* |
| OTU946 | No Hit | No Hit | No Hit | No Hit | No Hit | No Hit |

**Table S4. Top 30 OTUs highlighted in the biplots obtained from mmvec analysis.** The OTUS were retrieved from pair analysis between Healthy and ADNL groups.

| **kingdom** | **phylum** | **class** | **order** | **family** | **genus** | **Species** |
| --- | --- | --- | --- | --- | --- | --- |
| Bacteria | Actinobacteria | Actinobacteria | Corynebacteriales | Corynebacteriaceae | *Corynebacterium* | *Corynebacterium* sp |
| Bacteria | Firmicutes | Bacilli | Lactobacillales | Lactobacillaceae | *Lactobacillus* | *Lactobacillus iners* |
| Bacteria | Firmicutes | Clostridia | Clostridiales | Unclassified | Unclassified | Unclassified |
| Bacteria | Proteobacteria | Betaproteobacteria | Burkholderiales | Comamonadaceae | *Acidovorax* | *Acidovorax* sp |
| Bacteria | Proteobacteria | Alphaproteobacteria | Rhodobacterales | Rhodobacteraceae | *Paracoccus* | *Paracoccus* sp |
| Bacteria | Actinobacteria | Actinobacteria | Corynebacteriales | Corynebacteriaceae | *Corynebacterium* | *Corynebacterium urealyticum* |
| Bacteria | Bacteroidetes | Bacteroidia | Bacteroidales | Prevotellaceae | *Prevotella* | *Prevotella bivia* |
| Bacteria | Firmicutes | Bacilli | Bacillales | Staphylococcaceae | *Staphylococcus* | *Staphylococcus* sp |
| Bacteria | Firmicutes | Tissierellia | Tissierellales | Peptoniphilaceae | *Anaerococcus* | *Anaerococcus* sp |
| Bacteria | Firmicutes | Tissierellia | Tissierellales | Peptoniphilaceae | *Anaerococcus* | *Anaerococcus senegalensis* |
| Bacteria | Actinobacteria | Actinobacteria | Micrococcales | Micrococcaceae | *Micrococcus* | *Micrococcus* sp |
| Bacteria | Firmicutes | Unclassified | Unclassified | Unclassified | Unclassified | Unclassified |
| Bacteria | Actinobacteria | Actinobacteria | Micrococcales | Dermacoccaceae | *Dermacoccus* | *Dermacoccus* sp |
| Bacteria | Cyanobacteria | Unclassified | Unclassified | Unclassified | Unclassified | Unclassified |
| Bacteria | Proteobacteria | Gammaproteobacteria | Pseudomonadales | Pseudomonadaceae | *Pseudomonas* | *Pseudomonas* sp |
| Bacteria | Proteobacteria | Gammaproteobacteria | Pseudomonadales | Moraxellaceae | *Moraxella* | *Moraxella osloensis* |
| Bacteria | Firmicutes | Tissierellia | Tissierellales | Peptoniphilaceae | *Anaerococcus* | *Anaerococcus tetradius* |
| Bacteria | Actinobacteria | Actinobacteria | Corynebacteriales | Corynebacteriaceae | *Turicella* | *Turicella* sp |
| Bacteria | Proteobacteria | Gammaproteobacteria | Aeromonadales | Aeromonadaceae | *Aeromonas* | *Aeromonas caviae* |
| Bacteria | Actinobacteria | Actinobacteria | Corynebacteriales | Corynebacteriaceae | *Corynebacterium* | *Corynebacterium* sp |
| Bacteria | Firmicutes | Bacilli | Lactobacillales | Lactobacillaceae | *Lactobacillus* | *Lactobacillus crispatus* |
| Bacteria | Firmicutes | Bacilli | Lactobacillales | Lactobacillaceae | *Lactobacillus* | *Lactobacillus sakei* |
| Bacteria | Firmicutes | Bacilli | Lactobacillales | Lactobacillaceae | *Lactobacillus* | *Lactobacillus curvatus* |
| Bacteria | Actinobacteria | Actinobacteria | Corynebacteriales | Corynebacteriaceae | *Corynebacterium* | *Corynebacterium casei* |
| Bacteria | Actinobacteria | Actinobacteria | Micrococcales | Dermabacteraceae | *Brachybacterium* | *Brachybacterium paraconglomeratum* |
| Bacteria | Unclassified | Unclassified | Unclassified | Unclassified | Unclassified | Unclassified |
| Bacteria | Actinobacteria | Actinobacteria | Corynebacteriales | Corynebacteriaceae | *Corynebacterium* | *Corynebacterium auriscanis* |
| Bacteria | Proteobacteria | Alphaproteobacteria | Rhodobacterales | Rhodobacteraceae | *Paracoccus* | *Paracoccus* sp |
| Bacteria | Proteobacteria | Gammaproteobacteria | Pseudomonadales | Moraxellaceae | *Acinetobacter* | *Acinetobacter* sp |
| Bacteria | Actinobacteria | Actinobacteria | Micrococcales | Micrococcaceae | *Kocuria* | *Kocuria rhizophila* |

**Table S5. Top 30 OTUs highlighted in the biplots obtained from mmvec analysis.** The OTUS were retrieved from pair analysis between Healthy and ADL groups.

| **OTU** | **phylum** | **class** | **order** | **family** | **genus** | **species** |
| --- | --- | --- | --- | --- | --- | --- |
| OTU1035 | Proteobacteria | Alphaproteobacteria | Rhodospirillales | Acetobacteraceae | *Roseomonas* | *Roseomonas mucosa* |
| OTU1051 | Firmicutes | Bacilli | Bacillales | Staphylococcaceae | *Staphylococcus* | *Staphylococcus petrasii* |
| OTU130 | Proteobacteria | Alphaproteobacteria | Rhodobacterales | Rhodobacteraceae | *Paracoccus* | *Paracoccus sp* |
| OTU135 | Actinobacteria | Actinobacteria | Corynebacteriales | Corynebacteriaceae | *Corynebacterium* | *Corynebacterium urealyticum* |
| OTU1384 | Bacteroidetes | Flavobacteriia | Flavobacteriales | Flavobacteriaceae | *Haloanella* | *Haloanella gallinarum* |
| OTU1433 | Bacteroidetes | Bacteroidia | Bacteroidales | Prevotellaceae | *Prevotella* | *Prevotella bivia* |
| OTU1486 | Actinobacteria | Actinobacteria | Corynebacteriales | Corynebacteriaceae | *Corynebacterium* | *Corynebacterium massiliense* |
| OTU152 | Proteobacteria | Alphaproteobacteria | Rhodobacterales | Rhodobacteraceae | *Paracoccus* | *Paracoccus sp* |
| OTU1545 | Firmicutes | Bacilli | Bacillales | Staphylococcaceae | *Staphylococcus* | *Staphylococcus sp* |
| OTU1553 | Firmicutes | Bacilli | Lactobacillales | Streptococcaceae | *Streptococcus* | *Streptococcus thermophilus* |
| OTU1639 | Bacteroidetes | Bacteroidia | Bacteroidales | Prevotellaceae | *Prevotella* | *Prevotella sp* |
| OTU1678 | Firmicutes | Tissierellia | Tissierellales | Peptoniphilaceae | *Anaerococcus* | *Anaerococcus senegalensis* |
| OTU1827 | Firmicutes | Bacilli | Lactobacillales | Streptococcaceae | *Streptococcus* | *Streptococcus sp* |
| OTU189 | Actinobacteria | Actinobacteria | Micrococcales | Dermacoccaceae | *Dermacoccus* | *Dermacoccus sp* |
| OTU1955 | Proteobacteria | Gammaproteobacteria | Pseudomonadales | Moraxellaceae | *Moraxella* | *Moraxella osloensis* |
| OTU198 | Firmicutes | Bacilli | Lactobacillales | Streptococcaceae | *Streptococcus* | *Streptococcus sp* |
| OTU2126 | Actinobacteria | Actinobacteria | Corynebacteriales | Corynebacteriaceae | *Turicella* | *Turicella sp* |
| OTU230 | Actinobacteria | Actinobacteria | Corynebacteriales | Corynebacteriaceae | *Corynebacterium* | *Corynebacterium sp* |
| OTU245 | Proteobacteria | Betaproteobacteria | Neisseriales | Neisseriaceae | *Neisseria* | *Neisseria sp* |
| OTU25 | Cyanobacteria | Unclassified | Oscillatoriales | Unclassified | *Halospirulina* | *Halospirulina sp* |
| OTU277 | Firmicutes | Bacilli | Bacillales | Staphylococcaceae | *Staphylococcus* | *Staphylococcus aureus* |
| OTU363 | Firmicutes | Clostridia | Clostridiales | Peptostreptococcaceae | *Peptostreptococcus* | *Peptostreptococcus anaerobius* |
| OTU401 | Actinobacteria | Actinobacteria | Corynebacteriales | Corynebacteriaceae | *Corynebacterium* | *Corynebacterium casei* |
| OTU566 | Actinobacteria | Actinobacteria | Corynebacteriales | Corynebacteriaceae | *Corynebacterium* | *Corynebacterium auriscanis* |
| OTU645 | Firmicutes | Bacilli | Lactobacillales | Streptococcaceae | *Streptococcus* | *Streptococcus sp* |
| OTU682 | Actinobacteria | Actinobacteria | Corynebacteriales | Corynebacteriaceae | *Corynebacterium* | *Corynebacterium ureicelerivorans* |
| OTU759 | Actinobacteria | Actinobacteria | Corynebacteriales | Corynebacteriaceae | *Corynebacterium* | *Corynebacterium sp* |
| OTU872 | Actinobacteria | Actinobacteria | Micrococcales | Micrococcaceae | *Kocuria* | *Kocuria rhizophila* |
| OTU906 | Actinobacteria | Actinobacteria | Propionibacteriales | Propionibacteriaceae | *Propionibacterium* | *Propionibacterium sp* |
| OTU946 | No Hit | No Hit | No Hit | No Hit | No Hit | No Hit |

**References**

1. Bittremieux W, Avalon NE, Thomas SP, Kakhkhorov SA, Aksenov AA, Gomes PWP, Aceves CM, Rodríguez AMC, Gauglitz JM, Gerwick WH, Jarmusch AK, Kaddurah-Daouk RF, Kang KB, Kim HW, Kondić T, Mannochio-Russo H, Meehan MJ, Melnik AV, Nothias L-F, O’Donovan C, Panitchpakdi M, Petras D, Schmid R, Schymanski EL, van der Hooft JJJ, Weldon KC, Yang H, Zemlin J, Wang M, Dorrestein PC. 2022. Open Access Repository-Scale Propagated Nearest Neighbor Suspect Spectral Library for Untargeted Metabolomics. bioRxiv.
